# Pharmacological Evidences for Curcumin Neuroprotective Effects against Lead-Induced Neurodegeneration: Possible Role of Akt/GSK3 Signaling Pathway

**DOI:** 10.22037/ijpr.2020.1101210

**Published:** 2020

**Authors:** Negin Goudarzi, Sanaz Mohammad Valipour, Akram Nooritahneh, Majid Motaghinejad, Manijeh Motevalian, Sepideh Safari, Mina Gholami, Safieh Vatandour, Malak Hekmati

**Affiliations:** a *Department of Veterinary and Biomedical Science University of Minnesota, USA. *; b *Department of Pharmaceutics, Faculty of Pharmacy, Tabriz University of Medical Sciences, Tabriz, Iran* *.*; c *Department of Organic Chemistry, Faculty of Pharmaceutical Chemistry, Tehran Medical Sciences, Islamic Azad University, Tehran, Iran. *; d *Razi Drug Research Center, Iran University of Medical Sciences, Tehran, Iran. *; e *School of Medicine, Shahid Beheshti University of Medical Sciences, Tehran, Iran. *; f *Department of Animal science, Qaemshahr Branch, Islamic Azad University, Mazandaran, Iran.*

**Keywords:** Lead, Curcumin, Neurodegeneration, Akt, GSK

## Abstract

One of main herbal compounds with neuroprotective effects is curcumin. Lead poisoning cause neurodegeneration effect but its clear mechanism remains unknown. The current study evaluates the role of Akt/GSK3 signaling pathway in mediating the neuroprotective effects of curcumin against lead -induced neurodegeneration in rats. Sixty adult male rats were divided to: Group 1 and 2 receiving normal saline and drinking water containing 0.075% of lead acetate. Groups 3, 4, 5, and 6 were treated concurrently with lead acetate (0.075% in drinking water) and Curcumin (10, 20, 40, and 80 mg/kg I.P, respectively). Morris water maze (MWM) was used to evaluate cognitive activity, Hippocampal oxidative, anti-oxidant, as well as inflammatory and apoptotic factors and also Akt and GSK3 protein levels were studied. We found that lead poisoning disturbed the learning and memory and simultaneous treatment with Curcumin reduced the lead -induced cognition disturbances. In addition, lead acetate treatment increased lipid peroxidation and the levels of IL-1β, TNF-α , *Bax*, GSK3 (total and phosphorylated) while reducing reduced form of GSH, *Bcl-2*, and Akt3 (total and phosphorylated) levels in the hippocampus. Lead also reduced the activity of SOD, GPx, and GR in the hippocampus. In contrast, various doses of Curcumin attenuated lead -induced apoptosis, oxidative stress and inflammation; while elevating P-Akt and reduced of GSK3 levels. Thus, Curcumin via mediation of Akt/GSK3 signaling pathway confers neuroprotection against lead-induced neurodegeneration in hippocampus.

## Introduction

Poisoning with lead which is a heavy metal with neurotoxcity properties has been increased in recent years (1, 2). The consequences of chronic poisoning with lead and its biochemical and behavioral effects remain unclear (2, 3). Previous studies in recent years showed lead poising effects on brain function but its molecular effects and involved signaling pathways remain unclear 

(3, 4).Chronic poisoning with lead can induce behavioral changes such as cognition (learning and memory) impairment in rodent experimental model and also in human subjects (5, 6). Experimental and clinical studies have confirmed the potential effect of lead in neurodegeneration of some area of the brain such as the hippocampus and cortex which was responsible for cognition and motor activity (7-9). Previous studies have shown that lead poising can lead to production of apoptotic proteins like *Bax*, caspase, and therefore it causes DNA fragmentation in some brain region such as hippocampus and amygdala (9-11). Lead and other similar compounds can cause inflammation, oxidative stress, and mitochondrial dysfunction in brain cells, but involved signaling pathway remains unknown (7-9, 12).

In recent years, usage of herbal/natural compounds with therapeutic probability has been increased. Natural flavonoids and their derivatives are being extensively considered as therapeutics agents against neurodegenerative event which induced by some neurotoxic agent (13, 14). Curcumin (diferuloylleadane) is the most abundant component of turmeric, which is extracted from rhizomes of the plant curcuma longa. This non-nutritive yellow pigment is an established nutraceutical dietary phenol and thus of important medicinal and pharmacological value (15). Curcumin has been shown to be an neuroprotective agent and cognitive enhancer (13, 14). It exerts biological effects through its antioxidant, anti-inflammatory, anti-apoptotic, and immunomodulatory activity (14, 16). Curcumin treatment has shown to counteract oxidative stress, by reducing lipid peroxidation and improving the activity of antioxidant enzymes, and inflammation, by reducing TNF-α and IL-1β levels, in brain cells (14, 16, 17). All these properties may contribute to therapeutic potential efficacy of Curcumin in neurodegenerative event, but it’s exact mechanism remains unclear (15). Many previous works indicated that phosphatidylinositol 3-kinase (PI3K) can activate Akt (Protein Kinase B) in brain cells and by activation of this protein, glycogen synthase kinase 3 (GSK3), which is involved in neurodegeneration will be inhibited and cells protected from neurodegenerative effects of GSK3 (18, 19). Also previous works showed the role of Akt/GSK3 signaling pathway on cognitive activity (18), Akt is an important protein which plays a key role in synaptic and neural survival, neuroprotection and neural plasticity and supports the growth and survival of neurons. It is highly expressed in some brain areas that are known to regulate cognition and neuroprotection (20, 21). Based on mentioned studies it is suggested that curcumin may protect hippocampal neurons against lead induced-oxidative stress, inflammation, and apoptosis damage via regulation of Akt and GSK3β but this concept was not approved definitely. According to this concept and because of importance of Akt/GSK3 signaling pathways in modulation of neuroprotection and cognition performance, this study was designed to assess the role of these pathways in conferring neuroprotective effects of curcumin against lead induced oxidative stress , inflammation ,apoptosis and alterations in neurobehavioral parameters. This also can make a better understanding of curcumin and lead poising and mechanisms involved. 

## Experimental

*Animals*

Sixty adult Wistar male rats, weighing between 250–300 g, were purchased from lab house of Iran University of Medical Sciences. They were kept in an under controlled temperature room (22 ± 0.5 °C) with 12-h light/dark cycle and had free access to food and water. This manuscript was prepared from supplementary data which obtained from research project with ethical code: IR.IAU.PS.REC.1398.348. 

*Drug*

Curcumin and lead acetate were purchased from Sigma-Aldrich (USA) and dissolved freshly in normal saline just before administration. 

*Experimental design*

Group 1 (control group) were administrated with normal saline (0.2mL/rat, i.p) for 21 days and Group 2 (lead treatment) received drinking water containing 0.075% of lead acetate for 21 days. 

Groups 3, 4, and 5 concurrently were treated by drinking water containing 0.075% of lead acetate and curcumin with a dosage of 10, 20, 40, and 80 mg/kg, as intraperitoneally, respectively for 21 days. 

During the 17th and 21st day, Morris water maze (MWM) task, a standard behavioral method for evaluation of learning and memory, was done in the animals. After 22nd day all animals were sacrificed. Oxidative stress, inflammation and apoptosis were also evaluated in hippocampal tissues. Keeping in view the importance of Akt/GSK3 signaling in the effect of curcumin on lead-induced disturbances in the Akt/GSK3 signaling pathway was studied in hippocampal tissues. 

*Behavioral methods*

*morris water maze task(MWM* **)**

MWM apparatus includes a black colored circular tank, filled with water, 160 cm in diameter and 90 cm in height, which was fixed in the center of the experimental lab. This equipment was divided into four quadrants (North, East, West, and South) and was filled with water to the height of 50 cm. The operator stays in the North-East part of the room. A disk on the platform with 15 cm diameter, which was hidden, was located 1 cm beneath the surface of the water. In the first 4 days of the experiment, which called training procedure, mentioned platform was randomly inserted persistently in one of the quarter. An automated infrared tracking system (CCTV B/W camera, SBC-300 (P), Samsung Electronics Co, Ltd, Korea) recorded the position of the experimented animal in the tank. The camera was mounted 2.4 m above the surface of the water (22, 23). 

*Handling*

On the first day before the start of the experiment, all of the rats one by one were positioned in the tank filling with 40 centigrade degree water, and room temperature (25 ± 2 ºC) and the experimenter guided the rat to swim and to reach to the quarter in which the mentioned platform was placed. In our experiment, the platform was situated in South-East quarter of a tank (22, 23).

*Training procedure*

Some discriminate landmarks (such as a distinguish picture, window, door, etc.) as put in the extra maze in the room for spatial cues for learning about the platform’s position for animals. As mentioned above, the position of the platform was set up in the South-East quarter of the MWM tank with 25 cm distance from the edge of the tank, and 1 cm beneath the surface of the water. For evaluation of learning procedure, each rat experimented for 4 trials in a day for 4 days. Each animal was randomly located in from four quarters (North, East, West, and South) respectively. During the learning procedure if the rats found the platform within the 60 Sec, the trial would be automat closed by a computer, but if they could not reach and found the platform within 60 sec the trial automat would be stopped by the computer.In learning experiment, two parameters were evaluated. 

1. The time of escape latency characterized by time to find the hidden platform 

2. Traveled distance which was confirmed by the distance each animal spent to reach and find the hidden platform.

In memory assessment procedure, on the fifth day (probe day), the platform was removed and animal randomly was terrified of the water from one of the above-mentioned directions (almost East) and the percentage of presence of animal in target quarter (South-East quarter) was recorded and calculated (22-26).

*Mitochondrial preparations*

The animals were anesthetized using sodium thiopental (50 mg/kg, i.p) and the hippocampus was isolated from each rat. The isolated tissues were homogenized in cold homogenization buffer (25 mmol/L 4-morpholinepropanesulfonic acid, 400 mmol/L sucrose, 4 mmol/L magnesium chloride (MgCl_2_), 0.05 mmol/L ethylene glycol tetraacetic acid (EGTA), pH = 7.3) and the homogenized tissues were centrifuged at 450 × *g *for 10 min. The supernatants obtained were re-centrifuged at 12000 × *g* for 10 min. Finally, the sediments were re-suspended in homogenization buffer and stored at 0 °C. Total mitochondrial proteins in tissues were determined using a Dc protein assay kit (Bio-Rad). Briefly; Bradford reagent (1 part Bradford: 4 parts dH_2_O) was added to serial dilution series (0.1-1.0 mg/mL) of a known protein sample concentration; e.g., bovine serum albumin (BSA), dissolved in homogenization buffer. These serial dilution series were prepared and used for providing a standard curve. On the other hand 10, 15, 20, 25, and 30 μL of the protein extract (homogenized cell solutions) were added to multiple wells. Bradford reagent was also added to each well. The density of colors of all wells was read by the plate reader at 630 nm. Finally, by using the standard curve, protein quantity in the extracts was obtained. These homogenized cell solutions, containing mitochondria of hippocampal cells, were analyzed for the measurement of oxidative stress and inflammatory markers (23, 27-30). 

*Measurement of oxidative stress parameters*

*Determination of lipid peroxidation*

For assessment of lipid peroxidation, malondialdehyde (MDA) a natural by-product was assessed. Briefly, 100 μL of SDS lysis solution was added to wells containing (100 μL) of sample solution or MDA standard. After shaking and incubation of these wells, 250 μL of thiobarbituric acid (TBA) reagent was added to each well and incubated at 95 °C for 45-60 min. Next, the tubes were centrifuged at 1000 × g for 15 min and 300 μL of n-Butanol was added to 300 μL of the supernatant. Then, the tubes were centrifuged for 5 min at 10,000 × g. Finally, the absorbance was read at 532 nm and the results obtained were expressed as nmol/mg of protein (27, 30-32). 

*Determination of GSH (Glutathione) and GSSG (Glutathione disulfide)*

For measuring GSH (Glutathione) and GSSG (Glutathione disulfide) levels, 25 μL of the IX glutathione reductase solution and 25 μL of the IX NADPH solution were added to a 96-well plate containing a standard solution of glutathione or a sample of homogenized solution. Then, 50 μL of the IX Chromogen was added to each well and mixed vigorously. Finally, the absorbance was read at 405 nm for each GSSG/GSH standard and sample. Using the standard curve, the levels of GSSG/GSH were quantified and expressed as nmol/mg of protein (27, 30, 33). 

*Determination of manganese superoxide dismutase (MnSOD) activity*

The previously described leadod was used to assess SOD activity (27, 34). SOD activity was measured using the following equation:

*Determination of glutathione peroxidase (GPx) activity*

GPx activity was assessed as described previously (27, 34). It was measured based on a change in absorbance [ΔA340/min] by the following equation:

ΔA340/min = A340nm (Start) A340nm (Stop)/Reaction time (min), any change in the absorbance is directly proportional to GPx activity. 

GPx activity: ΔA340/min × Reaction volume (mL) × Dilution factor of the original sample/Extinction coefficient for NADPH at 340 nm× Volumes of the tested sample. Results were expressed as mU/mg protein (27, 34). 

*Determination of and glutathione reductase (GR) activity*

GR activity was assessed as described previously (27, 34, 35). It was measured based on a change in absorbance [ΔA340/min] by the following equation:

ΔA340/min= A340nm (Start) A340nm (Stop)/Reaction time (min), any change in the absorbance is directly proportional to GR activity. 

GR activity: ΔA340/min × Reaction volume (mL) × Dilution factor of the original sample/Extinction coefficient for NADPH at 340 nm× Volumes of the tested sample. Results were expressed as mU/mg protein (27, 35). 

*Determination of protein expression alteration*

Concentrations (expression of protein) of Akt or GSK3 (total and phosphorylated), TNF-α, IL-1β, *Bax*, and *Bcl-2* was measured in cell lysate of hippocampal tissue and for this part commercially available ELISA kit (Genzyme Diagnostics, Cambridge, U.S.A) was used. Briefly, the wells containing sheep anti-rat BDNF,CREB (total and phosphorylated), IL-1β and TNF-α polyclonal antibody (Sigma Chemical Co., Poole, and Dorset, UK) were washed three times with washing buffer (0.5 mol/L of Sodium chloride (NaCl), 2.5 mmol/L sodium dihydrogen phosphate (NaH_2_PO_4_), 7.5 mmol/L Na_2_HPO_4_, 0.1% Tween 20, pH 7.2). Then, 100 mL of 1% (w/v) ovalbumin (Sigma Chemical Co., Poole, Dorset, UK) solution was added to each well and incubated at 37 °C for 1 hr. Following three washes, 100 mL of samples and standards were added to each well and incubated at 48 °C for 20 h. After three washes, 100 mL of the biotinylated sheep anti-rat IL-1β or TNF-α antibody (1: 1000 dilutions in washing buffer containing 1% sheep serum, Sigma Chemical Co., Poole, and Dorset, UK) was added to each well. Next, after 1-hour incubation and three washes, 100 mL avidin-HRP (Dako Ltd, UK) (1:5000 dilution in wash buffer) was added to each well and the plate was incubated for 15 min. After washing three times, 100 mL of TMB substrate solution (Dako Ltd., UK) was added to each well and then incubated for 10 min at room temperature. Then, 100 mL of 1 mol/L H_2_SO_4_ was added and absorbance was read at 450 nm. The results were expressed as ng IL-1β/mL or TNF-α/mL or *Bax* and *Bcl-2 *of suspension of hippocampus tissues and about the Akt or GSK3 (total and phosphorylated) were reported as pg/mL of suspension of hippocampus tissues (36-39)

*Statistical analysis*

The data were analyzed by GraphPad PRISM v.6 Software and averaged in every experimental group and expressed as Means ± Standard error of the means (SEM). Then, the differences between control and treatment groups were evaluated by ANOVA. To evaluate the severity of the behaviors, the differences between averages in each group were compared using the Tukey test at a significant level of (*P *< 0.001). 

## Results

*Evaluation of escape latency and traveled distance during training days in the Morris water maze (MWM)*

**Figure 1:**
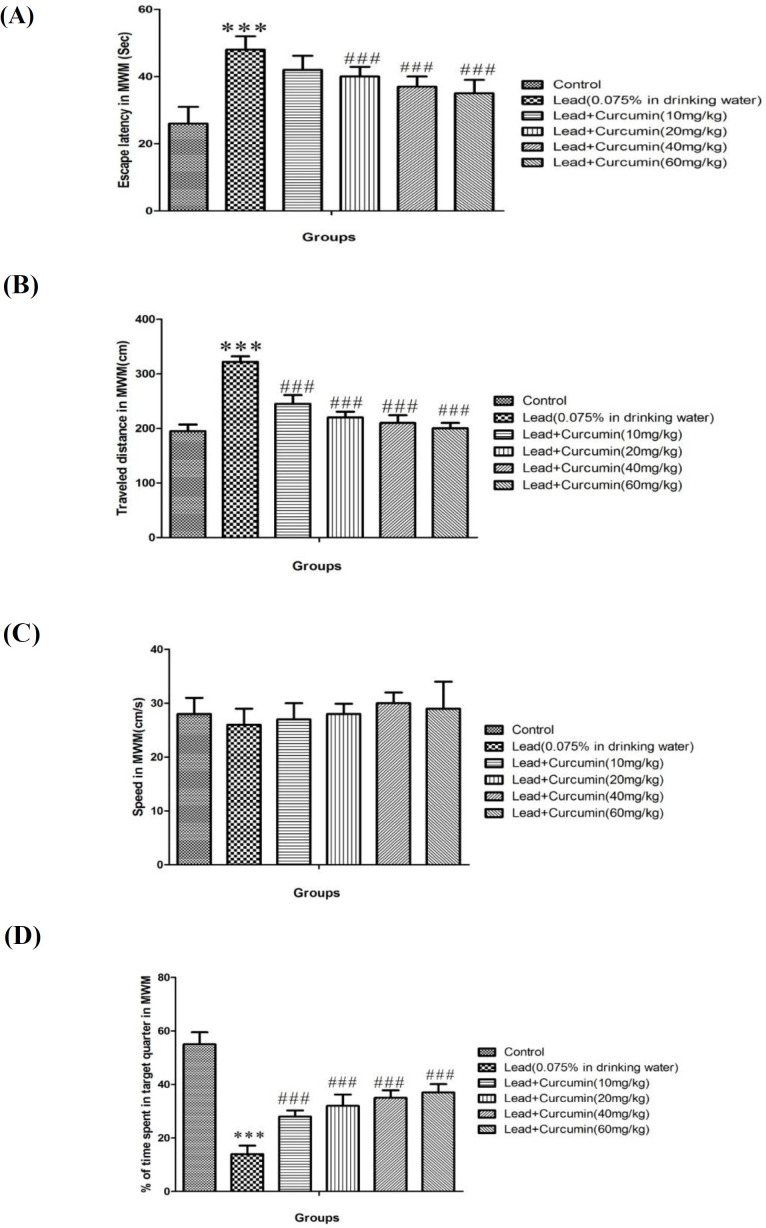
Average of escape latency (A), Average of traveled distance (B), Average of swimming speed (C) Percentages of time spent in target quarter in probe trial (D) in control group and groups under treatment by drinking water containing 0.075% of lead acetate in combination with Curcumin with doses of 10, 20, 40 and 60 mg/kg across all training days using Morris Water Maze (MWM) in rats. All data are expressed as Mean ± SEM (n = 10).

Lead poisoning cause significant increase of escape latency and traveled distance during four days training in the MWM when compared to control groups (*P *< 0.001) (Figure 1 A and B). While Curcumin inhibited lead-induced significant reduction in escape latency (with 20, 40 and 80 mg/kg of curcumin) and traveled distances (with all doses of curcumin) as opposed to lead treated group (*P *< 0.001) (Figure 1 A and B).

*Evaluation of swimming speed during training days*

The swimming speed was not altered during training trials in any of the animal groups (Figure 1C).

*Evaluation of percentage in target quarter in probe trial*

Lead poisoning, cause significant decrease in the percentage of the presence of animals in target quarter in comparison with control groups (*P *< 0.001) (Figure 1D). Also. Curcumin in all doses of could significantly diminish lead induced decrease in presence of animals in target quarter (*P*< 0.001) (Figure 1D).

*Effects of various doses of Curcumin on Lead-induced GSH/GSSG alterations*

LEAD (10 mg/kg) treatment markedly reduced the mitochondrial GSH content while increased the GSSG levels in comparison to the control group (*P *< 0.001). Conversely, high doses of Curcumin (40 and 80 mg/kg) improved the GSH-content and reduced the GSSG levels in LEAD-treated animals when compared to the positive controls (*P *< 0.001) (Table 1). 

*Effects of various doses of Curcumin on Lead-induced alteration in oxidative stress parameters*

**Figure 2.**
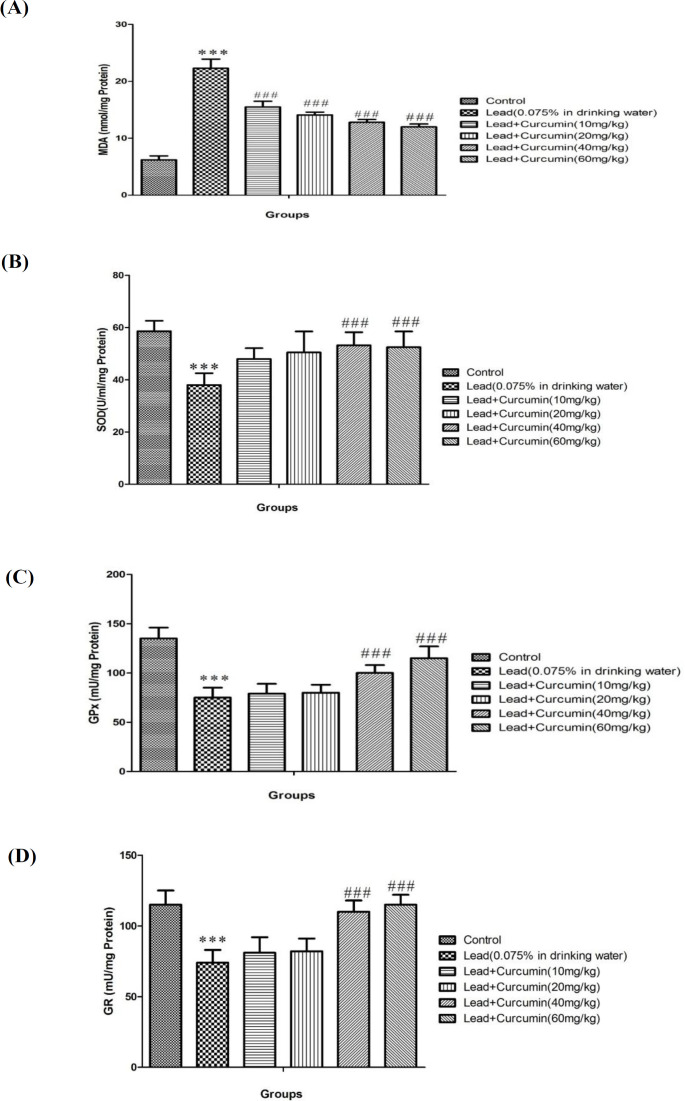
Effects of various doses of Curcumin (10, 20, 40 and 60 mg/kg) on Lead -induced lipid peroxidation (A), SOD activity (B), GPx activity (C) and GR activity (D) in rat isolated hippocampus

Poisoning with lead acetate significantly increased the MDA levels and reduced the SOD, GPx and GR activity when compared to the control group (*P *< 0.001) (Figure-2 A, B, C and D). Conversely**, **high doses of Curcumin inhibit the LEAD-induced increase in MDA levels (with all doses of curcumin) and decreases in SOD, GPx, and GR activity (with doses of 40 and 80 mg/kg of curcumin) (*P *< 0.001) (Figure 2A, B, C and D).

*Effects of various doses of Curcumin on Lead-induced rise inflammatory biomarker*

**Figure.3:**
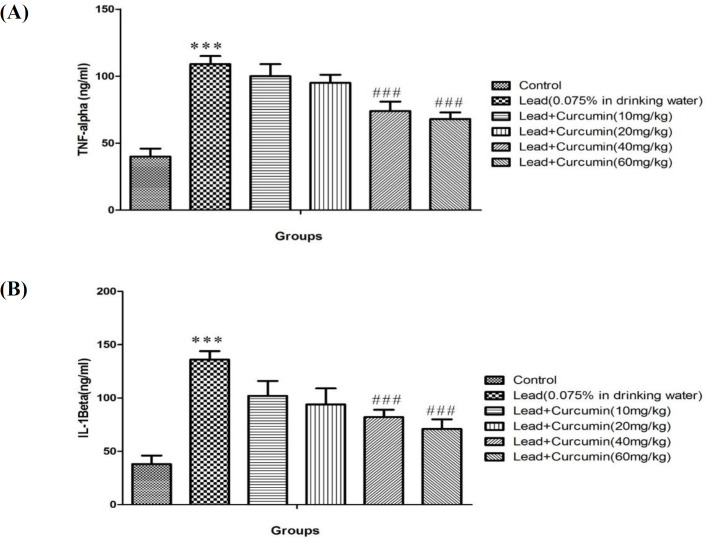
Effects of various doses of Curcumin (10, 20, 40 and 60 mg/kg) on Lead -induced alteration in TNF-α (A) and IL-1β (B) level in rat isolated hippocampus

Treatemnt of animal with lead acetate , causes significant elevation level of in IL-1β and TNF-α as compared to the control group (*P *< 0.001) (Figure 3 A and B). Conversely, high doses of Curcumin (40 and 80 mg/kg) prevented the lead-induced rise in level of in IL-1β and TNF-α when compared to lead only treated group (*P *< 0.001) (Figure 3 A and B).

*Effects of various doses of Curcumin on Lead-induced changes in Bax and Bcl-2 proteins level*

**Figure 4:**
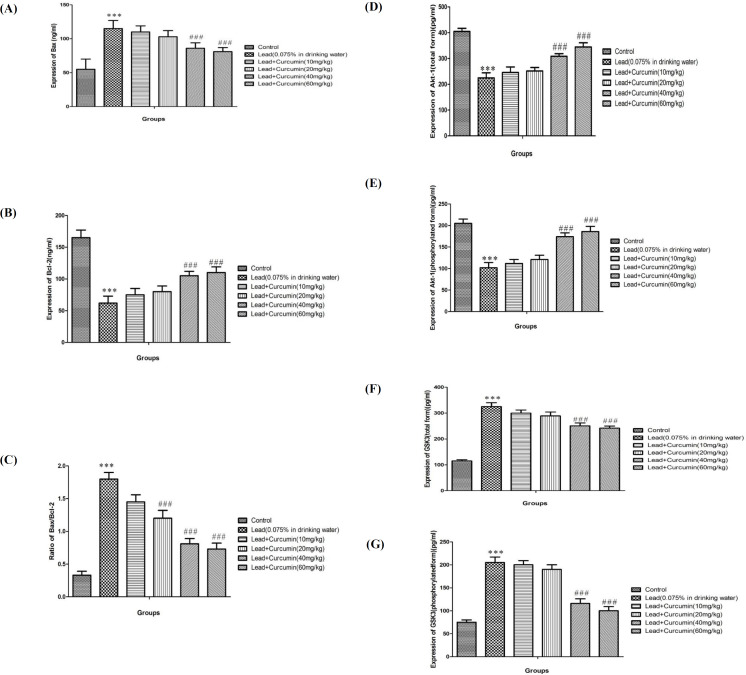
Effects of various doses of Curcumin (10, 20, 40 and 60 mg/kg) on lead-induced alteration of protein expression of *Bax* (A), *Bcl-2* (B), ratio of *Bax*/*Bcl-2* (C), total Akt (D), phosphorylated Akt (E), total GSK3 (F) and phosphorylated GSK3 (G) in rat isolated hippocampus.

Treatment of animal with lead acetate increased protein expression of *Bax* and reduced protein expression of *Bcl-2* when compared to the control group (*P *< 0.001). Conversely, high doses of Curcumin (40 and 80 mg/kg) improved *Bcl-2* protein expression while reduced *Bax* protein expression when compared to the positive controls (*P *< 0.001) (Figures 4 A, B and C).

*Effects of various doses of Curcumin on lead-induced alteration in protein expression of both forms of Akt and GSK3*

Lead poisoning markedly reduced the protein expression of Akt (total and phosphorylated), while increased the protein expression of GSK3 (total and phosphorylated) when compared to control group (*P *< 0.001) (Figure 4 D, E, F and G). Conversely, in lead tretad aniaml administartion of high doses of Curcumin (40 and 80 mg/kg) significantly improved the protein expression of Akt (total and phosphorylated) and decreased GSK3 (total and phosphorylated) when compared to the lead only treated group (Figure 4 D, E, F and G).

## Discussion

The results of current study demonstrate that various doses of curcumin can ameliorate lead-induced neuro-apoptosis, oxidative stress, and inflammation in the rat hippocampus. In addition, it indicates that the protective role of curcumin is mediated possibly via Akt/GSK3 signaling pathway.

Lead as a heavy metal carries a high potential for poising (4, 5). According to our study chronic poising with lead can increase escape latency and traveled distance in MWM, this data suggested that lead poising decrease learning activity. Also in probe day lead poising could decrease percentage of presence in target quarter in MWM. This data suggested that chronic administration of lead can decrease memory; this result confirms previous study results which indicated chronic lead poising decreased learning and memory in rats (3, 8, 40). Lead poising caused the damage of hippocampus cell in the brain and consequence of this phenomenon is cognition impairment (40, 41). According to our results curcumin at high doses (20, 40 and 80 mg/kg), could alter the lead-induced cognition impairment. Many previous studies indicate that Curcumin and other similar herbal compound can improve learning and memory (42, 43).

Our data indicated that lead poisoning increases hippocampal MDA level, whereas, curcumin treatment (10, 20, 40 and 80 mg/kg) attenuates lead-induced rise in lipid peroxidation in the brain. These results are similar to previous findings, which indicated lead-induced lipid peroxidation in the brain (43).According to these data, it seems that part of the destructive effects of lead is mediated through mitochondrial dysfunction and probably curcumin is somehow modulating this process (43-45). Furthermore, it has been indicated by previous work that curcumin exerts neuroprotective effects by inhibiting the formation of free radicals in neurodegenerative disorder (45), and the role of curcumin as a scavenger for free radicals is well-evident in this type of disorder (44, 45). Our results indicated that lead poisoning decreases mitochondrial GSH content while increasing GSSG level in the hippocampal tissues. Activation of glutathione form (GSH) to the toxic oxidized form (GSSG) by lead is a key change that can start and activate neurodegenerative signals in the brain (46, 47), and this mechanism causes harmful effect on glutathione cycle and consequently causes neural cell death which is consequences of lead poisoning (46, 47). Moreover, we found that various doses of curcumin, in all mentioned doses, increase GSH content, while reducing GSSG level in animals with lead consumption. These findings have also been reported already by previous studies indicating that curcumin, by modulation of glutathione circle ,can be therapeutically beneficial against neurodegenerative diseases as it promotes GSH formation (43, 44)

In our study, administration of lead decreased GPx, GR, and SOD activities in isolated hippocampal tissues, confirming the previous studies report about lead effects on diminishes of antioxidant defenses that may result in neurodegeneration (48, 49). It has been shown that GR is the key enzyme which responsible for converting the oxidized form of glutathione (GSH) to the reduced form of it (GSSG) (50).Thus, a lead-induced decrease in GR activity results in elevation of GSSG and reduction of GSH levels as observed in our results. Some novel reports showed that lead consumption causes mitochondrial dysfunction and inhibition of antioxidant enzyme activity in multiple cells, and these properties cause lead-induced degenerative effects on brain cells such as hippocampus (49). We detected that curcumin treatment dose-dependently recovers the activity of antioxidant enzymes. Curcumin by activating GR increases the conversion of GSSG to GSH and thus, protects the brain against lead-induced oxidative stress. Previous experimental studies have also established such anti-oxidative properties of curcumin in neurodegenerative disorder and diseases were mediated by increasing of GR and GPx activity (51, 52). In addition, our results confirmed the previous findings regarding the decrease in SOD activity following lead poisoning (53). Inconsistent with previous studies, treatment by curcumin was found to be effective in reversing this induction of reduction in SOD activity in the hippocampal tissues (54). We demonstrated that chronic lead poisoning significantly increases the level of pro-inflammatory cytokines like IL-β and TNF-α in the hippocampal tissue, whereas, curcumin in high doses (40 and 80 mg/kg) has a strong potential for suppressing lead-induced neuroinflammation in a dose-dependent manner. Our result is consistent with previous works which have reported the rise of pro-inflammatory cytokines following lead and other heavy metal poisoning. It has been proposed that lead-induced rise in inflammation is responsible for the neurodegenerative properties of lead (55). On the other way, curcumin has shown to have the potential therapeutic effects for management of neuroinflammation signaling cascades, thereby protecting the brain against inflammation and its damage (44, 45).

In addition to oxidative stress and inflammation evaluation, this study confirms lead-induced apoptosis in the hippocampus. According to the current study, lead consumption in animal increased the level of an apoptotic protein, *Bax*, while decreasing an anti-apoptotic protein, *Bcl-2*. This data is inconsistent with previous works which have been demonstrated that lead consumption can cause brain damage via activation of multiple apoptotic cascades (56).On the other hand, our results demonstrated the anti-apoptotic effect of curcumin against lead induced apoptosis, as indicated by reducing *Bax* and improved *Bcl-2* expressions in the hippocampus. Previous studies demonstrated that curcumin treatment attenuates cleaved caspase-3 and production of *Bax* and nuclear condensation resulting from some neurodegenerative disorder and disease (57).

The anti-inflammatory, anti-apoptotic, and anti-oxidative effects of curcumin have been previously reported (57), and were inconsistent with our work, but the involved signaling pathways remain unknown. In this regard, we evaluated the role of the Akt/GSK3 signaling pathway. Our data demonstrated that lead poisoning ameliorates Akt (total and phosphorylated) while increasing the GSK3 (total and phosphorylated) protein expression in the hippocampus. In contrast, curcumin treatment in high doses enhanced Akt (total and phosphorylated) and decreased GSK3 (total and phosphorylated) protein expression. Thus, it can be speculated that curcumin treatment restores Akt protein expression and by this effect inhibits GSK3 protein expression and protects the brain against lead-induced neurotoxicity. The P-Akt regulates over hundred target genes, especially genes implicated in neuronal regeneration, development, survival, and excitability, addiction, depression and cognition (58). In addition, dysregulation of Akt or upregulation of GSK3 transcriptional cascade has shown to induce oxidative stress, apoptosis, and neurodegeneration (59). Many previous molecular studies verified that the phosphorylated form of Akt has the main role in many herbal and chemical neuroprotective possessions (60). According to present data by lead poisoning , by activation of GSK3, some neurodegenerative events will occur in brain cells and some neurobehavioral disorders such as cognition impairment can be related to inhibition of Akt and activation of GSK3, which is involved in neurodegeneration (19). Also our data showed that curcumin can inhibit lead induced decreases of Akt protein level/expression in total and phosphorylated form, while it causes decreases of GSK3 in both forms in methamphetamine treated rats (61).It has been shown by many previous studies that curcumin cognitive enhancer effects against neurodegeneration were mediated by modulation of Akt/GSK3 and other similar signaling pathways (62). But the role of Akt/GSK3 in curcumin neurobehavioral and neurochemical changes had not clarified yet which according to the present findings, curcumin and other similar agents might act through these pathways (Akt/GSK3), and rescue cell survival and triggers neuroprotection. These novel results give us new insights in molecular effects of duloxetine in hippocampal cells. 

*** *P* < 0.001 *vs* control.

**###** *P* < 0.001 *vs* group under treatment by drinking water containing 0.075% of lead acetate.

All data are expressed as Mean ± SEM (n = 10).

*** *P*< 0.001 *vs* control.

All data are expressed as Mean ± SEM (n = 10).

*** *P*< 0.001 *vs* control

**###** *P*< 0.001 *vs* group under treatment by drinking water containing 0.075% of lead acetate.

All data are expressed as Mean ± SEM (n = 10).

*** *P*< 0.001 *vs* control

**###** *P*< 0.001 *vs* group under treatment by drinking water containing 0.075% of lead acetate.

**Table 1:** Effects of various doses of Curcumin on mitochondrial GSH and GSSG content in lead treated rats

**Group**	**GSH (nmol/mg protein)**	**GSSG (nmol/mg protein)**	**GSH/GSSG**
Control group	69.5 ± 6.1	0.76 ± 0.4	92
Lead (0.075% in drinking water)	34.7 ± 6.2^a^	6.9 ± 1.1^a^	4.9^a^
Lead +Curcumin(10mg/kg)	42.4 ± 1.9	4.9 ± 0.04	8.5
Lead +Curcumin(20mg/kg)	53.3 ± 5^ b^	4.1 ± 0.11^ b^	12.9^ b^
Lead +Curcumin(40mg/kg)	60.3 ± 2.3^ b^	3.6 ± 0.12^ b^	16.6^ b^
Lead +Curcumin(60mg/kg)	62.2 ± 3.2^b^	2.9 ± 0.3^b^	21.9^b^

All data are presented as mean ± SEM, n = 10.

^a^ *P *< 0.001 *vs*. control group.

^b^ *P *< 0.001 *vs*. Lead treated group.

## Conclusion

For the first time, the results of the current study indicates that curcumin treatment, possibly via stimulation of Akt/GSK3 signaling pathway, can decline lead-induced apoptosis, oxidative stress, inflammation and cognition impairment and can be act as neuroprotective agent against lead induced neurodegeneration. However, further studies regarding human dosage and toxicity and also possible therapeutic effects in human subject are necessary. 
